# Modulation of Ca^2+^ Signaling by Anti-apoptotic B-Cell Lymphoma 2 Proteins at the Endoplasmic Reticulum–Mitochondrial Interface

**DOI:** 10.3389/fonc.2017.00075

**Published:** 2017-05-03

**Authors:** Tim Vervliet, Eva Clerix, Bruno Seitaj, Hristina Ivanova, Giovanni Monaco, Geert Bultynck

**Affiliations:** ^1^Laboratory of Molecular and Cellular Signaling, Department of Cellular and Molecular Medicine, KU Leuven, Leuven, Belgium

**Keywords:** endoplasmic reticulum–mitochondria contact sites, Ca^2+^-transport systems, apoptosis, autophagy, mitochondrial bio energetics, IP_3_ receptors, voltage-dependent anion channels, Bcl-2

## Abstract

Mitochondria are important regulators of cell death and cell survival. Mitochondrial Ca^2+^ levels are critically involved in both of these processes. On the one hand, excessive mitochondrial Ca^2+^ leads to Ca^2+^-induced mitochondrial outer membrane permeabilization and thus apoptosis. On the other hand, mitochondria need Ca^2+^ in order to efficiently fuel the tricarboxylic acid cycle and maintain adequate mitochondrial bioenergetics. For obtaining this Ca^2+^, the mitochondria are largely dependent on close contact sites with the endoplasmic reticulum (ER), the so-called mitochondria-associated ER membranes. There, the inositol 1,4,5-trisphosphate receptors are responsible for the Ca^2+^ release from the ER. It comes as no surprise that this Ca^2+^ release from the ER and the subsequent Ca^2+^ uptake at the mitochondria are finely regulated. Cancer cells often modulate ER-Ca^2+^ transfer to the mitochondria in order to promote cell survival and to inhibit cell death. Important regulators of these Ca^2+^ signals and the onset of cancer are the B-cell lymphoma 2 (Bcl-2) family of proteins. An increasing number of reports highlight the ability of these Bcl-2-protein family members to finely regulate Ca^2+^ transfer from ER to mitochondria both in healthy cells and in cancer. In this review, we focus on recent insights into the dynamic regulation of ER–mitochondrial Ca^2+^ fluxes by Bcl-2-family members and how this impacts cell survival, cell death and mitochondrial energy production.

## Introduction

Ca^2+^ signaling plays important roles in a vast amount of cell physiological processes (1). In cancer cells, Ca^2+^ signaling is altered to promote mitochondrial bioenergetics, cell proliferation, migration, and survival while inhibiting cell death (2–6). The involvement of Ca^2+^ signaling in the development of cancer and consequently the potential of Ca^2+^ signaling as a target for treatment is becoming increasingly apparent (5–11). In cancer cells, proteins involved in Ca^2+^ signaling have been reported to have differential expression profiles compared to healthy cells (12–15). In addition, an increasing number of proto-oncogenes and tumor suppressors impact Ca^2+^-signaling pathways by directly modulating intracellular Ca^2+^-transport systems with critical functions in cell survival and cell death (16–19).

An important Ca^2+^-signaling pathway involved in both cell death and cell survival is the transfer of Ca^2+^ from the endoplasmic reticulum (ER) to the mitochondria (20). These Ca^2+^ transfers occur at the so-called mitochondria-associated ER membranes (MAMs), which are close contact sites between the ER and the mitochondria (21). A continuous small Ca^2+^ transfer to the mitochondria is necessary to maintain proper energy production (22). Ca^2+^ is required by several enzymes of the tricarboxylic acid (TCA) cycle (like pyruvate dehydrogenase, isocitrate dehydrogenase and α-ketoglutarate) to promote NADH and ATP production (23). Besides this, Ca^2+^ also modulates the ATP synthase complex V and the adenine nucleotide translocator (24). In addition to this mitochondrial pathway, pro-survival Ca^2+^ oscillations activate calcineurin, which in turn dephosphorylates the nuclear factor of activated T-cells (NFAT), conferring its translocation into the nucleus (25). Here, NFAT triggers the transcription of genes involved in cell proliferation. In contrast, large Ca^2+^ transfers from the ER to the mitochondria may result in both Ca^2+^-induced mitochondrial outer membrane permeabilization (MOMP) and opening of the mitochondrial permeability transition pore (mPTP), the latter formed by dimers of the F_0_F_1_ ATP synthase (4, 26, 27). In this process, Ca^2+^ overload in the mitochondria triggers cardiolipin oxidation, resulting in the disassembly of the respiratory chain complex 2 (also known as succinate dehydrogenase), subsequently leading to excessive reactive oxygen species (ROS) production (28). Mitochondrial produced ROS can open the mPTP, ultimately leading to MOMP. At the level of the ER, the inositol 1,4,5-trisphosphate (IP_3_) receptor (IP_3_R) (29) is an important intracellular Ca^2+^-release channel involved in these Ca^2+^ transfers, whereas at the mitochondria, the voltage-dependent anion channel (VDAC) (at the outer mitochondrial membrane) (30) and the mitochondrial Ca^2+^ uniporter (MCU) (at the inner mitochondrial membrane) (31, 32) are important for transporting Ca^2+^ into the mitochondrial matrix.

The B-cell lymphoma 2 (Bcl-2)-protein family, consisting of both anti- and pro-apoptotic members, is critically involved in regulating cell death and survival (33–36). Dysregulated expression and function of Bcl-2 proteins have been not only implicated in oncogenesis but also represent an “Achilles’ heel” in cancer cells that can be exploited by the use of Bcl-2 inhibitors (37–39). Anti-apoptotic Bcl-2 proteins (like Bcl-2, Bcl-X_L_ and Mcl-1) have been extensively described to inhibit apoptosis by neutralizing the pro-apoptotic Bcl-2-family members (like Bax, Bak, Bim, Bid, etc.). The mechanism involves binding of the Bcl-2 homology (BH) 3 domains of the pro-apoptotic proteins to the hydrophobic cleft formed by the BH1, BH2 and BH3 domains of the anti-apoptotic members, thereby inhibiting cell death (40). A recently developed class of compounds, so-called BH3-mimetic drugs (40–42), is able to compete with pro-apoptotic Bcl-2-family members for the hydrophobic cleft of the anti-apoptotic Bcl-2-family members. Hence, BH3-mimetics alleviate the inhibition of Bax and Bak by the anti-apoptotic Bcl-2-family members, effectively killing cancer cells that are dependent on anti-apoptotic Bcl-2 proteins for their survival. In addition to this, the BH4 domain of Bcl-2 also contributes to the interaction with Bax *via* a site that is distinct from Bax’s BH3 domain (43). Moreover, the isolated BH4 domain, delivered as a stapled peptide, neutralized the pro-apoptotic activity of Bim-derived BH3 peptides by restricting Bax’s conformational change (44).

**Figure 1:**
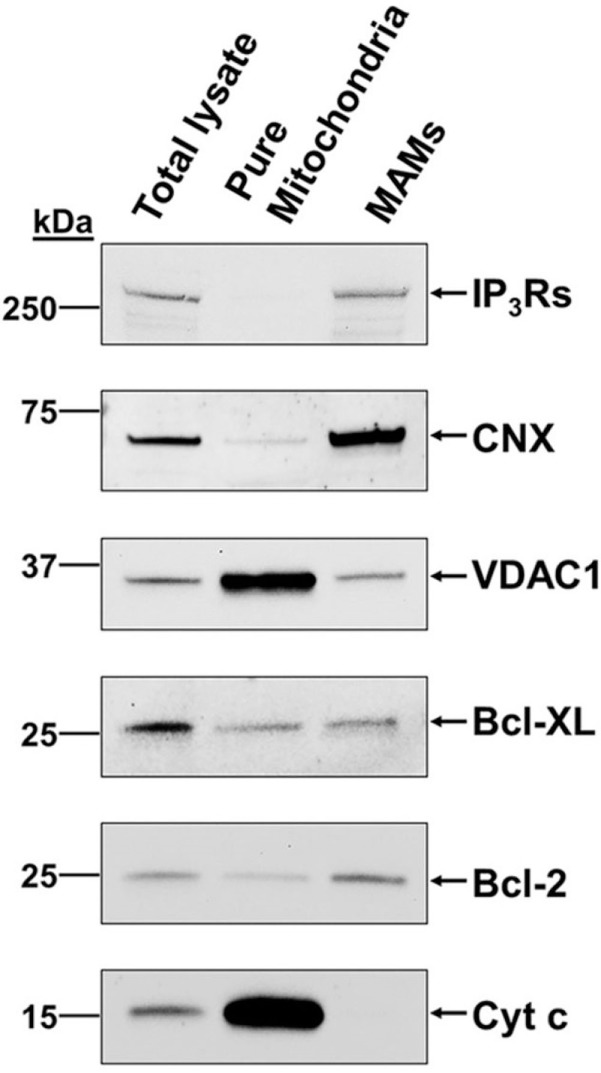
**B-cell lymphoma (Bcl)-2 and Bcl-X_L_ and their targets in Ca^2+^ signaling, inositol 1,4,5-trisphosphate receptor (IP_3_R) and voltage-dependent anion channel 1 (VDAC1), are present in the mitochondria-associated endoplasmic reticulum membranes (MAMs)**. Representative immunoblots showing the presence of VDAC1, IP_3_Rs, Bcl-2, and Bcl-X_L_ in the MAMs of MEFs. Calnexin (CNX) and cytochrome *c* (Cyt *c*) served as specific MAMs and mitochondrial markers, respectively. These data were originally published in *Journal of Biological Chemistry* with following reference: Monaco et al. (45). © The American Society for Biochemistry and Molecular Biology. Authors of articles in Journal of Biological Chemistry have the rights to reuse their own material and are automatically granted a permission to reuse figures from their articles in future works. The original results have been produced by Dr. Alex van Vliet in the laboratory of Prof. Patrizia Agostinis (KU Leuven, Belgium).

**Figure 2:**
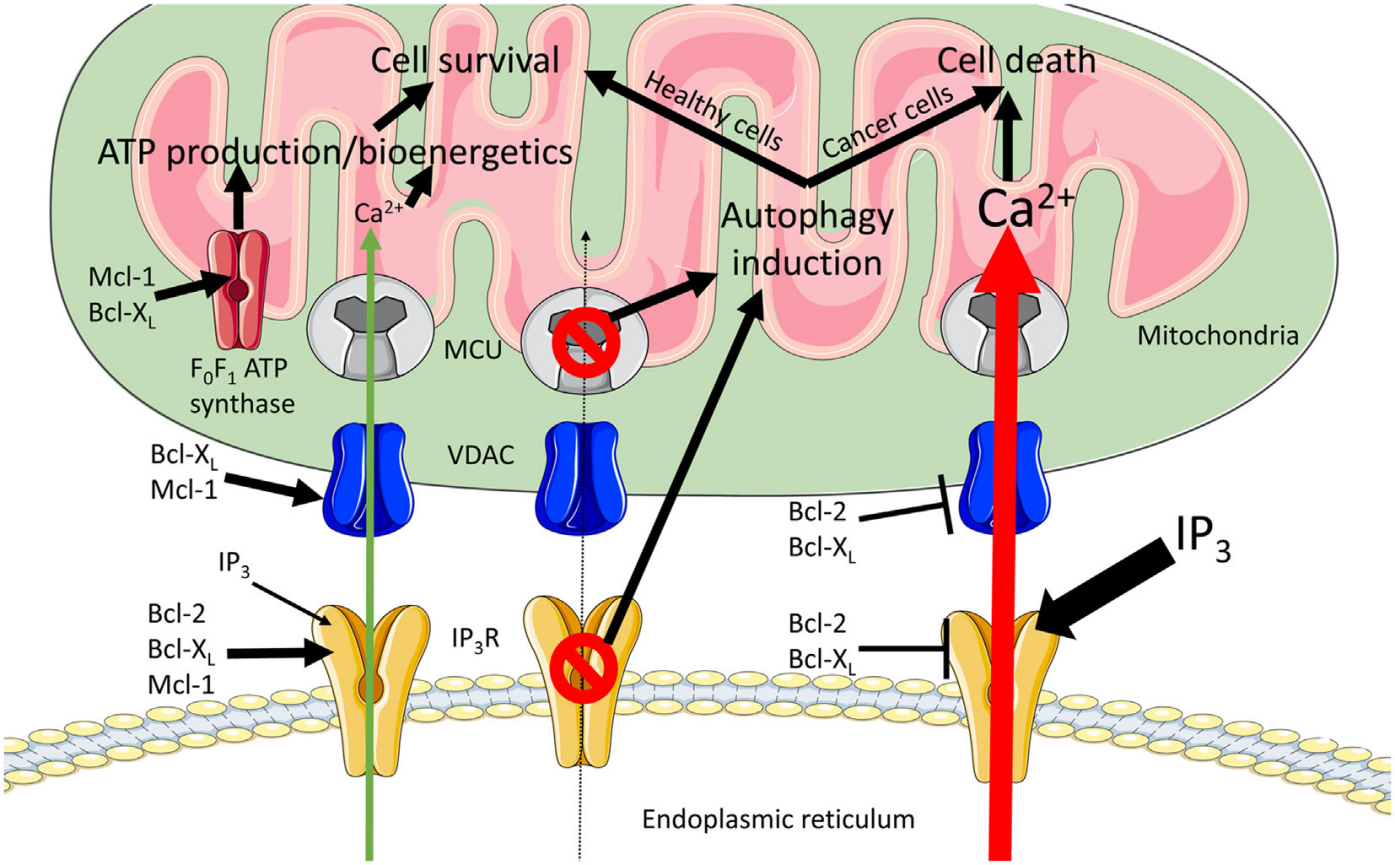
**Modulation of endoplasmic reticulum (ER) to mitochondrial Ca^2+^ transfers by anti-apoptotic B-cell lymphoma (Bcl)-2 proteins**. ER to mitochondrial Ca^2+^ transfers are critical for the regulation of cell death and cell survival decisions. In order to fuel the tricarboxylic acid (TCA) cycle, a continuous influx of Ca^2+^ into the mitochondria is required (green arrow), thereby promoting cell survival. Excessive mitochondrial Ca^2+^ uptake leads to Ca^2+^-induced mitochondrial outer membrane permeabilization (MOMP) and cell death (red arrow). The anti-apoptotic side of the Bcl-2-protein family regulates these Ca^2+^ transfers at both organelles. During pro-survival Ca^2+^ signaling at the ER, Bcl-2, Bcl-X_L_, and Mcl-1 modulate inositol 1,4,5-trisphosphate receptor (IP_3_R)-mediated Ca^2+^ release to generate Ca^2+^ oscillations. At the mitochondria, Bcl-X_L_ and Mcl-1 can increase voltage-dependent anion channel 1 (VDAC1)-mediated Ca^2+^ uptake. Combining the effects at the two organelles results in an efficient and finely regulated Ca^2+^ uptake at the mitochondria, which increases mitochondrial bioenergetics and promotes cell survival. In addition, Mcl-1 and Bcl-X_L_ target the F_0_F_1_ ATP synthase, thereby regulating ATP-production. During pro-death signaling, Bcl-2 and Bcl-X_L_ can inhibit both pro-apoptotic Ca^2+^ release from the IP_3_R and the Ca^2+^ uptake into the mitochondria *via* VDAC. Finally, abolishing ER to mitochondrial Ca^2+^ transfers by either blocking IP_3_Rs or knocking down the mitochondrial Ca^2+^ uniporter (MCU) induces autophagy. When this is coupled to decreased cell proliferation (healthy cells), this increase in autophagy may rescue the cell. However, when proliferation is not halted (cancer cells) this results in cell death.

Anti-apoptotic Bcl-2 proteins are also known to regulate ER to mitochondrial Ca^2+^ signaling at both organelles, and several Bcl-2-family members, including Bcl-2 and Bcl-X_L_, are present in the MAMs (45, 46) (Figure 1). At the ER, anti-apoptotic Bcl-2, Bcl-X_L_ and Mcl-1 promote pro-survival IP_3_R-mediated Ca^2+^ oscillations, enhancing cell proliferation and mitochondrial energy production (47–49). Bcl-2 (and Bcl-X_L_ at high concentrations) also inhibits excessive pro-apoptotic IP_3_R-mediated Ca^2+^ release (50–53), thereby preventing Ca^2+^-induced MOMP. At the mitochondrial side of the MAMs, anti-apoptotic Bcl-2 and Bcl-X_L_ proteins inhibit VDAC1-mediated Ca^2+^ uptake in the mitochondria (45, 54, 55). However, also stimulatory roles of Bcl-2-family members on VDAC1-mediated mitochondrial Ca^2+^ transfer have been described, thereby maintaining adequate mitochondrial Ca^2+^ levels that promote survival and mitochondrial bioenergetics (56, 57). Besides IP_3_Rs and VDAC, anti-apoptotic Bcl-2-family members also regulate other members of the Ca^2+^ toolkit at different locations in the cell [extensively reviewed in Ref. (33)]. Mcl-1, located at the inner mitochondrial membrane, was also shown to be crucial for normal mitochondrial bioenergetics by regulating the assembly of the F_0_F_1_ ATP synthase oligomers (58). Finally, the F_0_F_1_ ATP synthase emerged as a target for anti-apoptotic Bcl-X_L_, allowing the direct regulation of ATP production (59, 60). In this review, we will focus on recent insights into the dynamic regulation of ER–mitochondrial Ca^2+^ fluxes, the involvement of anti-apoptotic Bcl-2-family members and how this impacts cell survival, cell death, and mitochondrial energy production (Figure 2), three important aspects of cancer development.

## ER Side of the MAMs

ER Ca^2+^ release is an important determinant for cell survival by regulating mitochondrial bioenergetics and for cell death *via* promoting mPTP opening. In most cells, including cancer cells, the IP_3_R is an important intracellular Ca^2+^-release channel responsible for Ca^2+^ release from the ER. Cancer cells have developed several ways to modulate IP_3_R-mediated Ca^2+^ release, among which Bcl-2-dependent regulation.

### IP_3_R

A continuous Ca^2+^ flux from the ER to the mitochondria is necessary in order to maintain normal energy production. At the ER, the IP_3_R is responsible for the Ca^2+^ release and is present at the MAMs (Figure 1). Inhibition of the IP_3_R and thus of the continuous Ca^2+^ transfer to the mitochondria was already shown to result in the induction of autophagy, thereby managing the decrease in mitochondrial energy production (22). New findings emerged, showing that cancer cells are addicted to constitutive IP_3_R-driven Ca^2+^ transfer to the mitochondria (61, 62). Similar to normal/non-tumorigenic cells, cancer cells increase their autophagic flux upon IP_3_R inhibition in order to cope with the loss of Ca^2+^ influx into the mitochondria and subsequent reduction in energy production. However, in normal cells, the increased autophagy is accompanied by a decrease in the proliferation rate at the G1/S checkpoint (63), addressing the decreased availability of mitochondrial substrates for biosynthetic pathways of nucleosides and other cellular building blocks. In this way, cells may survive until normal Ca^2+^ transfer to the mitochondria is restored. In cancer cells, this increase in autophagy is not accompanied by a reduction in cell proliferation, likely due to a loss of the link between the monitoring of the mitochondrial health and the G1/S checkpoint. As such, these malignant cells proceed through the cell cycle without the necessary pool of nucleosides, resulting in a mitotic catastrophe and necrotic cell death.

Anti-apoptotic Bcl-2-family members have been shown to regulate the IP_3_R. Both inhibitory (50–52) and stimulatory (47, 49) effects, largely dependent on the Bcl-2-family member involved (64, 65) and the strength of IP_3_R activation (25, 53), have been described. As such, it was reported that in T-cell models, Bcl-2 suppresses IP_3_R-mediated Ca^2+^ release generated by strong T-cell receptor stimulation, thereby preventing excessive Ca^2+^ transfer into the mitochondria. This interaction occurs *via* Bcl-2’s BH4 domain and a stretch of 20 amino acids in the central coupling domain of the IP_3_R (52, 64). Peptides derived from this amino acid stretch were able to disrupt IP_3_R/Bcl-2 complexes in several cell types and models, thereby augmenting cell death in response to apoptotic triggers that act through Ca^2+^ signaling (25, 66). The efficient IP_3_R inhibition by anti-apoptotic Bcl-2 critically depended on the presence of Bcl-2’s C-terminal transmembrane domain, which interacted with the C-terminal domain of the IP_3_R channel (67). Bcl-2 lacking its transmembrane domain failed to inhibit IP_3_R-mediated Ca^2+^ release and to suppress Ca^2+^-dependent apoptosis in an *in cellulo* context. In contrast, the hydrophobic cleft of Bcl-2, responsible for scaffolding pro-apoptotic family members, was dispensable for IP_3_R binding and inhibition.

Bcl-2, Bcl-X_L_ and Mcl-1 were reported to sensitize the IP_3_R to low levels of IP_3_ in order to promote pro-survival Ca^2+^ oscillations, thereby feeding Ca^2+^ into the mitochondria to maintain adequate mitochondrial bioenergetics (47–49). The interaction of Bcl-2-family members with the C-terminus of the IP_3_R has been proposed as the underlying molecular mechanism for generating these Ca^2+^ oscillations (68). Recently, the mechanism underlying IP_3_R sensitization by Bcl-X_L_ has been identified with a prominent role for its hydrophobic cleft (53). Two BH3-like domains were identified in the C-terminus of IP_3_Rs. Indeed, in contrast to Bcl-2, for which its hydrophobic cleft was shown to be dispensable for IP_3_R modulation, Bcl-X_L_ *via* its hydrophobic cleft could target, with different affinities, both BH3-like domains present in the C-terminal region of the IP_3_R. At low concentrations, Bcl-X_L_ increased the open probability of the IP_3_R in response to low levels of IP_3_ by simultaneous binding to both BH3-like domains. Similar to Bcl-2, high Bcl-X_L_ concentrations were able to inhibit IP_3_R-mediated Ca^2+^ release in response to strong IP_3_R stimulation. The “dual” interaction with the BH3-like domain which conferred the highest affinity toward Bcl-X_L_ as well as the region in the coupling domain of the IP_3_R targeted by Bcl-2’s BH4 domain, was important for IP_3_R inhibition by Bcl-XL. Binding of Bcl-X_L_ to the coupling domain appeared with much lower affinity than the binding to the C-terminal tail, which is in line with our previous study that focused on the binding efficiency of Bcl-2 *versus* Bcl-X_L_ for both IP_3_R domains (48). This may indicate that moderate levels of Bcl-X_L_ will most likely operate in IP_3_R-sensitizing modus and thus will promote Ca^2+^ oscillations, whereas high levels of Bcl-X_L_ will be needed to operate in IP_3_R-inhibiting modus. Finally, binding of Bcl-X_L_ to both BH3-like domains is involved in maintaining cell viability and in protecting cells from stress inducers. These molecular results substantiate the previously observed sensitization of the IP_3_R by Bcl-X_L_ (68), resulting in pro-survival Ca^2+^ oscillations, and underscore the importance of this interaction for cell viability.

The role of Bcl-X_L_ in modulating IP_3_R-mediated Ca^2+^ release in order to promote mitochondrial bioenergetics was recently further highlighted (69). The authors showed that Bcl-X_L_ interacts with IP_3_R3 at the MAMs, where it increased Ca^2+^ transfer into the mitochondria, thereby enhancing TCA cycling. Upon ER-stress induction, Bcl-X_L_ translocated more to the MAMs, where the subsequent facilitation of Ca^2+^ transfer to the mitochondria and thus increased energy production helped the cells cope with the induced ER stress. This further highlights that Bcl-X_L_ exerts its protective effects against stress inducers in large part *via* modulating Ca^2+^ signaling.

## Mitochondrial Side of the MAMs

Cancer cells are highly dependent on the mitochondria for their energy production. For sustaining this energy production, adequate control of mitochondrial Ca^2+^ levels is important. Anti-apoptotic Bcl-2 proteins are known regulators of this mitochondrial Ca^2+^ influx, thereby regulating mitochondrial bioenergetics. In addition, the F_0_F_1_ ATP synthase has also been identified as a target for anti-apoptotic Bcl-2-family members, thereby directly linking them to the production of ATP (58–60).

### VDAC

The large conductance channel VDAC, of which three isoforms are known to exist, is located at the outer mitochondrial membranes (30). At the MAMs, VDAC is physically linked to the IP_3_R *via* molecular tethers like the chaperone protein, glucose-regulated protein 75, allowing efficient Ca^2+^ transfer from the ER into the mitochondria (70). Close regulation of mitochondrial Ca^2+^ uptake *via* VDAC is critical for maintaining mitochondrial energy production. Anti-apoptotic Bcl-2-family members are known to modulate this mitochondrial Ca^2+^ transfer through interactions with VDAC. Both Bcl-2 and Bcl-X_L_ have been reported to inhibit VDAC1-mediated Ca^2+^ uptake into the mitochondria, thereby protecting cells from Ca^2+^-induced MOMP (45, 54, 55, 71). The BH4 domain of Bcl-X_L_, but not the one of Bcl-2, was sufficient to bind to VDAC1 and to directly inhibit VDAC1 single-channel activity (45). Although different regions of Bcl-2 and Bcl-X_L_ seem to be involved in this interaction, both anti-apoptotic proteins target the N-terminus of VDAC1. Introducing VDAC1’s N-terminal into cells was shown to inhibit both Bcl-2’s and Bcl-X_L_’s anti-apoptotic function, illustrating that VDAC1 could be a target for anti-cancer drugs (54, 71–73). However, at the level of the BH4 domains, the N-terminal peptide of VDAC1 could only counteract the inhibitory action of Bcl-X_L_’s, but not that of Bcl-2’s BH4 domain. The BH4 domain of Bcl-2 also suppressed agonist-induced mitochondrial Ca^2+^ uptake and staurosporine-induced cell death, but acted through inhibition of IP_3_Rs, since IP_3_R-derived peptides were able to alleviate the inhibitory effects of Bcl-2’s, but not those of Bcl-X_L_’s BH4 domain (45).

Although the interaction of Bcl-X_L_ with VDAC1 is well established, the impact of Bcl-X_L_ on VDAC1’s functional properties may be dichotomous. Besides inhibiting VDAC1 (45, 54), Bcl-X_L_ has been reported to enhance VDAC1 activity. Bcl-X_L_ knockout MEF cells displayed a reduced VDAC1-mediated Ca^2+^ uptake in the mitochondria compared with the wild-type MEF cells (56). Similarly, N-terminal peptides derived from VDAC1 that disrupt Bcl-X_L_ binding to VDAC1 could also antagonize mitochondrial Ca^2+^ uptake in wild-type MEF cells, while these peptides lacked any effect in Bcl-X_L_-deficient MEF cells. While differences in experimental conditions may underlie the seemingly contrasting observations, these results indicate that Bcl-X_L_ might have a dual impact on VDAC1’s Ca^2+^-flux properties dependent on VDAC1’s function as a pro-survival or pro-death protein. Hence, Bcl-X_L_ could stimulate basal pro-survival and inhibit excessive pro-apoptotic VDAC1-mediated mitochondrial Ca^2+^ transfer, thereby fine-tuning mitochondrial Ca^2+^ handling according to cellular needs, with respect to cell fate decisions. The molecular basis for these opposite effects of Bcl-X_L_ on VDAC1 remains poorly understood.

Mcl-1 has also been shown to positively regulate VDAC in non-small cell lung carcinoma cells (57). In these cancer cells, Mcl-1 interacted with VDAC, with a pronounced role for its N-terminus, thereby increasing mitochondrial Ca^2+^ uptake, resulting in increased ROS production and cell migration. Disrupting the Mcl-1/VDAC interaction utilizing N-terminal VDAC-derived peptides could inhibit ROS production and cell migration. The importance of Mcl-1 at the mitochondria was further underscored by a recent study concerning different Mcl-1 splice variants (74). In this study, the increased expression of the short pro-apoptotic Mcl-1 isoform resulted in increased mitochondrial fusion *via* a reduced Mcl-1-dependent recruitment of dynamin-related protein 1 to the mitochondria. This was accompanied by hyperpolarization of the mitochondrial potential and increased mitochondrial Ca^2+^ uptake, thereby increasing susceptibility to apoptotic stimuli. Whether this increase in mitochondrial Ca^2+^ uptake was also mediated through the interaction with VDAC was not evaluated. Nevertheless, it would be interesting to assess whether the short pro-apoptotic Mcl-1 isoform would shift VDAC-mediated mitochondrial Ca^2+^ uptake toward more pro-apoptotic levels in comparison to the long pro-survival Mcl-1 isoform.

### F_0_F_1_ ATP Synthase

In cultured hippocampal neurons, Bcl-X_L_ was shown to be present at the inner mitochondrial membranes, where it directly targets the β-subunit of the F_0_F_1_ ATP synthase (59, 60). The interaction stabilized the mitochondrial membrane potential *via* the closure of a membrane leak pathway. This increased the enzymatic activity of the F_0_F_1_ ATP synthase, thereby promoting ATP production during neural activity. In addition, the interaction seems to occur *via* Bcl-X_L_’s hydrophobic cleft, since ABT-737 could reverse the effects of Bcl-X_L_ on the F_0_F_1_ ATP synthase. Recently, this process was further explored and was shown to be important for neuronal survival (75). In response to excitotoxic stimuli, cyclin B1 and cyclin-dependent kinase 1 (CdK1) accumulated in the mitochondria. There, the cyclin B1-Cdk1 complex phosphorylated Bcl-X_L_, leading to its dissociation from the ATP-synthase. This led to decreased ATP synthesis and production of ROS species, resulting in the inhibition of respiratory chain complex I, mitochondrial dysfunction, and potentially neuronal death.

## Potential Therapeutic Opportunities

### Promoting ER–Mitochondrial Ca^2+^ Transfer

Many chemotherapeutics trigger intracellular Ca^2+^ release from the ER, causing, or at least contributing to, mitochondrial Ca^2+^ overload. This Ca^2+^ release is often considered as a nonspecific side effect of the drug, but in many cases, it contributes to obtain maximal therapeutic effects (76). Moreover, recent studies have unraveled the molecular mechanisms underlying the impact of chemotherapeutics and photodynamic therapy on intracellular Ca^2+^ homeostasis (18, 77, 78). These anti-cancer regimens caused the accumulation of the tumor suppressor p53 at the ER membranes, where it enhanced sarco/endoplasmic reticulum Ca^2+^-ATPase (SERCA) 2b activity. The effects were independent of the transcriptional roles of p53. Recruitment of p53 at the ER augmented the Ca^2+^ filling state of the ER stores, increasing the susceptibility to apoptotic stimuli and the likelihood for mitochondrial Ca^2+^ overload. Cells deficient in p53 did not display this effect and were resistant to chemotherapy. This resistance could be overcome by SERCA and/or MCU overexpression.

### Inhibiting ER–Mitochondrial Ca^2+^ Transfer

The therapeutic potential of dampening Ca^2+^ transfer from ER to mitochondria has recently been proposed as an anti-cancer strategy (9, 61). It was shown that dysregulation of the ER to mitochondrial Ca^2+^ transfer *via* inhibition of IP_3_Rs, results in the induction of autophagy in both cancer and normal cells. However, when this increase in autophagy is not accompanied by a halt in proliferation, the cancer cells will die mainly through necrosis. This could prove to be a very specific way of eliminating cancer cells by effectively turning the increased proliferative capacity of cancer cells against themselves, whereas healthy cells can cope with this loss of Ca^2+^ transfer to the mitochondria. A major challenge will be to selectively target the Ca^2+^ transfer into the mitochondria without affecting global Ca^2+^ signaling and to mainly limit the effect of IP_3_R-inhibiting drugs to the malignant cells.

### Antagonizing Anti-apoptotic Bcl-2 Proteins

Major efforts have been dedicated towards the development of BH3-mimetic drugs, which target the hydrophobic cleft of anti-apoptotic Bcl-2-family members. The first generation of BH3 mimetics (ABT-737 and ABT-263) inhibited both Bcl-2 and Bcl-X_L_, resulting in severe side effects related to thrombocytopenia due to the dependence of thrombocytes on Bcl-X_L_ for their survival (41, 79). More recently, a Bcl-2-selective BH3-mimetic inhibitor was developed, namely ABT-199/venetoclax, which is a very promising anti-cancer drug that has been approved for the treatment of chronic lymphocytic leukemia (80). Whether these BH3-mimetic drugs also influence the ability of anti-apoptotic Bcl-2-family members to modulate intracellular Ca^2+^ release is less well understood, although some recent studies aimed to address this. With the identification of the two BH3-like domains at the C-terminus of the IP_3_R, the ABT-737 compound was shown to disrupt the binding of Bcl-X_L_ to the C-terminus of the IP_3_R, thereby abolishing both the stimulatory and inhibitory effects of Bcl-X_L_ on IP_3_R-mediated Ca^2+^ release (53). However, the contribution of Ca^2+^ signaling to ABT-737-induced cell death requires further investigation, since ABT-737 could cause cell death in primary chronic lymphocytic leukemia cells without inducing elevations in intracellular [Ca^2+^] (81).

Besides a direct impact on IP_3_R/Bcl-X_L_ complexes, ABT-737 has also been proposed to modulate the sensitivity of cancer cells to chemotherapy *via* a mechanism that involves remodeling of ER–mitochondrial contact sites (82). As such, cisplatin-resistant ovarian cancer cells could be re-sensitized to cisplatin by ABT-737. This drug increased ER–mitochondrial contact sites, thereby increasing cisplatin-induced elevations in mitochondrial Ca^2+^. When co-applied with cisplatin in cholangiocarcinoma cells, ABT-737 has been shown to induce mitochondrial fragmentation and mitophagy, resulting in cell death, whereas cisplatin alone induced mitochondrial hyperfusion, potentially underlying cell-death resistance (83). The combined ABT-737/cisplatin treatment led to a decreased Mcl-1 and an increased Bax expression. Interestingly, Mcl-1 has recently been shown to be implicated in controlling mitochondrial dynamics (74).

Consistent with the lack of contribution of Bcl-2’s hydrophobic cleft to the interaction with and regulation of IP_3_R, IP_3_R/Bcl-2-protein complexes and IP_3_R inhibition by Bcl-2 were resistant to ABT199/venetoclax treatment (67). Acute addition of ABT199/venetoclax to a variety of permeabilized and intact cell systems did neither trigger Ca^2+^ release by itself nor directly affected ER-located Ca^2+^-uptake and -release systems. Related to this, ABT199/venetoclax-induced apoptosis in Bcl-2-dependent cancer cells appeared to occur independently of intracellular Ca^2+^ overload (67, 84). However, the inhibition of Bcl-2 by BH3-mimetics has been reported to result in a rapid impairment of mitochondrial oxidative phosphorylation (85). This may underlie the increased sensitivity of Bcl-2-dependent cancer cells to ABT199/venetoclax in the presence of the intracellular Ca^2+^ buffer, BAPTA-AM (67, 84).

Over the years, it has become clear that Bcl-2 inhibition *via* targeting its BH4 domain has potential as an effective anti-cancer treatment (38, 86–88). Targeting Bcl-2/IP_3_R complex with Bcl-2/IP_3_ receptor disrupter-2 (BIRD-2), a stabilized TAT-linked peptide containing the 20 amino acids that represent the Bcl-2 interaction motif of IP_3_Rs, triggers intracellular Ca^2+^ overload and apoptotic cell death in a variety of cancer cell models, including chronic lymphocytic leukemia (81), diffuse large B-cell lymphoma (89), multiple myeloma, follicular lymphoma (90), and small-cell lung carcinoma (91). The cell death could be suppressed by buffering intracellular Ca^2+^ and by inhibiting IP_3_R activity (81, 89). Very recently, a small molecule (BDA-366) that targets the BH4 domain of Bcl-2 has been developed and shown to be effective in lung cancers and multiple myeloma (92, 93). The mechanism involved a conformational switch in Bcl-2 that turned it from a pro-survival to a pro-death protein by exposing its BH3 domain. A decrease in Bcl-2 phosphorylation may contribute to this pro-apoptotic switch induced by BDA-366. BDA-366 also impaired IP_3_R/Bcl-2 complex formation and raised cytosolic Ca^2+^ levels, although further work is needed to determine the contribution of Ca^2+^ signaling to BDA-366-induced cell death in cancer cells.

Mcl-1 gene amplifications are frequently found in many types of cancer (94). Very recently, an Mcl-1 inhibitor (S63845) targeting Mcl-1’s hydrophobic cleft has been developed (95). This compound was shown to be very specific for Mcl-1, well-tolerated by animal models and efficient at triggering cell death in Mcl-1-dependent tumor cells. As the regulation of VDAC1 by Mcl-1 also stimulates cancer cell migration (57), Mcl-1 inhibitors may not only be useful to eliminate Mcl-1-dependent cancers by provoking cell death but also by counteracting metastasis. However, at this point, it is not clear whether these Mcl-1 inhibitors can disrupt VDAC1/Mcl-1 complex formation.

## Conclusions

Ca^2+^ transfer from ER to mitochondria is important for maintaining proper energy production and balance between cell survival and cell death. The anti-apoptotic Bcl-2-family members regulate these Ca^2+^ transfers at the level of the ER as well as of the mitochondria by directly targeting Ca^2+^-transport systems located at the ER and mitochondria. Moreover, the molecular determinants underlying the complex formation between the Bcl-2 proteins and these systems are emerging as a hot topic, which allows the development of strategies and tools to interfere with the Bcl-2 protein-mediated control of Ca^2+^-signaling. These mechanisms also appear to be exploited by cancer cells to promote survival and mitochondrial bioenergetics, to contribute to cell-death resistance and control metastasis. Thus, targeting the Ca^2+^-modulating abilities of Bcl-2 proteins may offer novel anti-cancer strategies. In addition to this, Ca^2+^ signaling might contribute to the cell-death properties of recently developed Bcl-2 inhibitors, including BH3-mimetics and BH4-domain antagonists.

## Author Contributions

TV and GB drafted the manuscript. All authors critically read, amended, and/or corrected the manuscript. TV made the figures.

## Conflict of Interest Statement

The authors declare that the research was conducted in the absence of any commercial or financial relationships that could be construed as a potential conflict of interest.
